# An Empirical Comparison of Information-Theoretic Criteria in Estimating the Number of Independent Components of fMRI Data

**DOI:** 10.1371/journal.pone.0029274

**Published:** 2011-12-27

**Authors:** Mingqi Hui, Juan Li, Xiaotong Wen, Li Yao, Zhiying Long

**Affiliations:** 1 National Key Laboratory of Cognitive Neuroscience and Learning, Beijing Normal University, Beijing, China; 2 J. Crayton Pruitt Family Department of Biomedical Engineering, University of Florida, Gainesville, Florida, United States of America; 3 College of Information Science and Technology, Beijing Normal University, Beijing, China; Cuban Neuroscience Center, Cuba

## Abstract

**Background:**

Independent Component Analysis (ICA) has been widely applied to the analysis of fMRI data. Accurate estimation of the number of independent components of fMRI data is critical to reduce over/under fitting. Although various methods based on Information Theoretic Criteria (ITC) have been used to estimate the intrinsic dimension of fMRI data, the relative performance of different ITC in the context of the ICA model hasn't been fully investigated, especially considering the properties of fMRI data. The present study explores and evaluates the performance of various ITC for the fMRI data with varied white noise levels, colored noise levels, temporal data sizes and spatial smoothness degrees.

**Methodology:**

Both simulated data and real fMRI data with varied Gaussian white noise levels, first-order auto-regressive (AR(1)) noise levels, temporal data sizes and spatial smoothness degrees were carried out to deeply explore and evaluate the performance of different traditional ITC.

**Principal Findings:**

Results indicate that the performance of ITCs depends on the noise level, temporal data size and spatial smoothness of fMRI data. 1) High white noise levels may lead to underestimation of all criteria and MDL/BIC has the severest underestimation at the higher Gaussian white noise level. 2) Colored noise may result in overestimation that can be intensified by the increase of AR(1) coefficient rather than the SD of AR(1) noise and MDL/BIC shows the least overestimation. 3) Larger temporal data size will be better for estimation for the model of white noise but tends to cause severer overestimation for the model of AR(1) noise. 4) Spatial smoothing will result in overestimation in both noise models.

**Conclusions:**

1) None of ITC is perfect for all fMRI data due to its complicated noise structure. 2) If there is only white noise in data, AIC is preferred when the noise level is high and otherwise, Laplace approximation is a better choice. 3) When colored noise exists in data, MDL/BIC outperforms the other criteria.

## Introduction

Functional magnetic resonance imaging (fMRI) technique has been successfully used to investigate cognitive functions of human brain by multivariate methods. Among various multivariate methods, spatial independent component analysis (sICA, but referred to ICA in this study) has been demonstrated to be a promising technique to explore spatially independently distributed neural networks from fMRI data without any prior information [1], [2].

Based on an important hypothesis that the detected signals are linear combinations of statically independent source signals, the ICA model can be expressed by
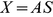
(1)Where 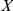 is an M×N matrix consisting of the raw fMRI data. M is the number of scans while N is the number of voxels. 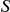 is a K×N matrix whose rows represent the spatially independent components and K is the number of total independent components. 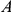 is an M×K mixing matrix. Each column of matrix 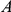 represents the time course of the corresponding independent components.

The basic goal of ICA is to estimate the spatially independent components 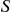 and the mixing matrix 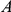. However, due to the high temporal dimensionality and high noise level of fMRI data, it would be very likely to over-fit the data [3] and result in splitting one component into two or more if ICA is applied on the full temporal dimension [4]. Therefore, the number of spatially independent components is often assumed to be less than the temporal dimension of fMRI data. A lower dimensional subspace containing the informative sources is usually identified by principle component analysis (PCA) prior to ICA. However, it is essential to estimate an appropriate dimension of the signal subspace in fMRI data before performing PCA. Underestimation may result in mixing various components [1], [5], [6], [7] and missing some valuable information while overestimation can result in splitting the true independent component [4], [7], [8], decreasing the stability of independent component estimates and making the interpretation of the ICA results difficult [9]. For example, Ma et al evaluated the ability of sICA to capture resting state functional connectivity with the number of independent components ranging from 2 to 30 and demonstrated that the result of ICA was affected if this number was too small [10].

Several methods based on ITC have been demonstrated to be attractive for model order selection in signal processing including Akaike's information criterion(AIC) [11], Kullback–Leibler information criterion(KIC) [12], the minimum description length (MDL) criterion [13], Bayesian information criterion (BIC) [14]and a Laplace approximation to Bayesian Criterion based on model evidence [15]. Among these criteria, AIC is an inconsistent estimator that tends, asymptotically, to overestimate the number of signals [16]. KIC tends to outperform AIC in that it is a consistent estimator and has less over-estimating than AIC [12]. MDL and BIC are consistent estimators [17], [18]. In the case of large sample, BIC can be regarded as an approximation of MDL despite being derived in an independent manner [13], [14]. There have been some classical comparisons of ITC. Some studies have mostly focused on comparisons between AIC and BIC in the context of the general linear model. Results of these studies have demonstrated that BIC is consistent and performs poorly in small samples whereas AIC is not consistent and performs relatively well in small sample [18], [19]. Liavas et al studied the influence of the distribution of the noise and signal eigenvalues on AIC and MDL's behavior [20]. Moreover, Fishler et al. (2002) investigated the performance of BIC in the blind source separation and suggested that BIC performed poorly at small sample sizes, but improved with increasing sample size [21].

Recently, many studies have attempted to apply ITC to estimate the number of independent components of fMRI data. For instance, the average of AIC and MDL estimates was used to determine the number of components in fMRI data [22]. BIC and Laplace approximation were also applied to fMRI data [4], [23]. Moreover, considering the spatial and temporal dependency of fMRI data, several improved methods were proposed to estimate the dimension of fMRI data [9], [24], [25]. Despite widespread use of ITC, there have been few empirical investigations of the performance of ITC in the context of ICA model. Even fewer investigations have fully compared or examined the performance of ITC with regard to the properties of fMRI data in particular. Comprehensively investigating the performance of different ITC in estimating the dimension of fMRI data is valuable to provide an insight into the selection of different ITC for fMRI data. The purpose of the current study was to empirically evaluate and compare the performance of ITC in the context of ICA model. Of particular interest was how these different criteria performed in estimating the number of independent components of fMRI data with different properties. The noises underlying real fMRI data includes Gaussian/white noise, such as thermal noise [26], and colored noise due to low-frequency physiological fluctuation [27]. Moreover, it has been demonstrated that the colored noises in fMRI data can become ‘white’ when a ‘whitening’ filter is applied [27]. Therefore, it is essential to investigate the impact of Gaussian/white noise and colored noise in fMRI data on the estimation of independent components by ITC. For colored noise, both the simple Auto-Regressive (AR) models or related Auto-Regressive and Moving Average (ARMA) models have been used to model autocorrelation of noise of fMRI data [28], [29], [30]. Although a higher order AR model may be better for time series of voxels that have a strong correlation structure, such as brain-stem voxels, ventricular voxels and larger vessels [29], the AR(1) noise model appears to work well for water phantom data and for preprocessed fMRI data with motion artifacts corrected and signal drifts removed [24]. In this paper, both the simulated and real fMRI data with varied Gaussian white noise levels, AR(1) noise levels, temporal data sizes and spatial smoothness degrees were used to deeply explore and compare the performance of the traditional ITC including AIC, KIC, MDL, BIC and Laplace approximation. Results show the performance of ITC is dependent on the standard deviation of Gaussian white noise, auto-correlation coefficient of AR(1) noise, temporal data size and spatial smoothness of the fMRI data. Moreover, the present study not only demonstrates some conclusions of previous studies, but also reveals some new information regarding the performance of different ITC. Some suggestions about how to choose a proper ITC according to the properties of fMRI data are given at the end of the paper.

## Methods

### Ethics Statement

The human fMRI experiment conducted in this study was approved by the Institutional Review Board of Beijing Normal University (BNU) Imaging Center for Brain Research, National Key Laboratory of Cognitive Neuroscience. The subjects gave written informed consent.

### Information-Theoretic criteria

The estimation of the number of independent component of fMRI data can be regarded as an issue of model order selection. Given a set of N observations 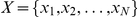 and a family of models, ITC aims at selecting the model that best fits the data. Suppose 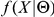 is the probability distribution of 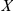, while 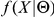 is an estimation of 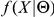. The AIC criterion is to select the model that gives the minimum Kullback-Leibler distance, defined by [11]

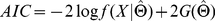
(2)Here 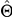 is the maximum likelihood estimate of the parameter vector 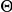. The first term is the maximum likelihood of the observations 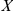 given the model parameter estimates and 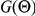 is a bias correction term to make the 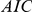 an unbiased estimate of the mean Kullback-Leibler distance between the modeled density 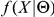 and the estimated density 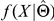.

Based on Akaike's work, Rissanen proposed to select the model that yields the minimum description length (MDL) given by [13]

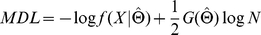
(3)Note that apart from a factor of 2, the first term is identical to the corresponding one in the AIC, while the second term has an extra factor of 0.5log 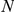 where 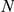 is the size of sample.

Wax and Kailath developed the order selection formulations of AIC and MDL based on the assumption of i.i.d Gaussian noise with zero mean and equal variance [16], expressed as Eqs. (4–5)
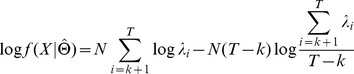
(4)

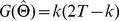
(5)Where 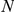 is the sample size. 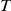 is the original dimension of the multivariate data. 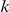 is the candidate order and 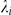
s are the eigenvalues of sample covariance matrix of the observations.

In 1999, Cavanaugh proposed a model selection criteria named as KIC which served as an asymptotically unbiased estimator of a variant of the Kullback's symmetric divergence between the true model and a fitted approximated model [12]

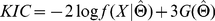
(6)Because maximum likelihood estimators can be obtained as large-sample limits of the Bayes estimators, Schwartz proposed to select the model that yielded the maximum posterior probability based on the assumption that each competing model could be assigned a prior probability [14]. The posterior probability is given by
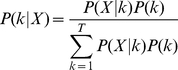
(7)Using Laplace's approximation, the posterior probability can be described by [15].
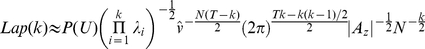
(8)Where
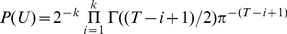
(9)

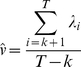
(10)

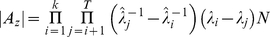
(11)where 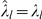 for 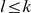 and 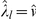 otherwise. Usually, log (Lap(k)) is used instead of Lap(k).

A simplification of Laplace's method is the BIC approximation. This approximation drops all terms which do not grow with N and can be simplified as [14], [15], [23]

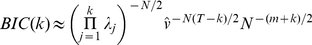
(12)


### Simulations

In this section, simulated fMRI data were generated to assess the performance of different ITCs by varying Gaussian white noise level, colored noise level, temporal data size and spatial smoothness of the simulated data. AIC, KIC, MDL, BIC and Laplace approximation were applied to each simulated dataset to estimate the number of independent components.

### White noise model

**Figure 1:**
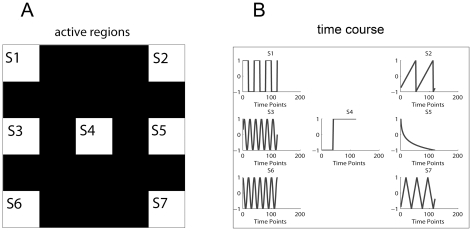
Seven simulated sources. A) The actived spatial regions. B) The corresponding time courses.

All simulated data in the following simulations were generated in a similar way. A two-dimensional 200×200 matrix with each pixel's intensity of 100 was duplicated T times, one for each time point. T was determined by each simulation. Gaussian noises with zero mean and specific standard deviation (SD) were added to all pixels at every time point to simulate system noises. As shown in Fig. 1A, seven white rectangular regions of interest (ROIs) were constructed over this matrix. The time courses added to the corresponding ROIs were shown in Fig. 1B. Each simulated experiment in each condition was repeated 50 times and the means of the 50 estimations of ITCs were obtained.

a) Effect of Gaussian Noise Level

The number of time points T was set to 120. The SD of Gaussian noise varied from 0.2 to 5 with an increase of 0.4.

b) Effect of temporal data size

Three Gaussian noise levels were set to SD = 1, 2 and 3. At each noise level, the number of time points T varied from 40 to 170 with an increase of 10.

c) Effect of spatial smoothness

The temporal data size T was 120. The noise levels were set to SD = 1, 2, 3. Each simulated data was spatially smoothed with a Gaussian filter. The full weight at half maximum (FWHM) of the Gaussian kernel of the filter was changed from 0.5 to 3 pixels.

### AR(1) noise model

The following simulated data were generated in the same way as above, except that colored noise rather than Gaussian white noise was added to all pixels at every time point. Colored noise was generated based on AR(1) model with the form of Eq. (13).
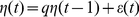
(13)Where q is the AR(1) coefficient and 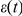 is a random variable with Gaussian distribution having a zero mean and specific SD. Both q and SD of AR(1) noise are factors to be investigated. Each simulated experiment in each condition was also repeated 50 times and the means of the 50 estimations of ITCs were obtained.

a) Effect of AR(1) coefficient

In investigation of the AR coefficient, the number of time points T was set to 120. Three AR(1) noise levels were set to SD = 1, 2 and 3. At each noise level, the AR(1) coefficient q varied from 0.05 to 0.95 with an increase of 0.05.

b) Effect of SD of AR(1) noise

The number of time points T was set to 120. Three AR(1) coefficient levels were set to q = 0.1, 0.5 and 0.9. The SD of AR(1) noise varied from 0.2 to 5 with an increase of 0.2.

c) Effect of temporal data size

Three AR(1) coefficient levels were set to q = 0.1, 0.5 and 0.9. The SD of AR(1) noise was set to 1. At each AR(1) coefficient level, the number of time points T varied from 80 to 320 with an increase of 40.

d) Effect of spatial smoothness

In this section, the temporal data size T was 120. The SD of AR(1) noise was set to 1. Three AR(1) coefficient levels were set to q = 0.1, 0.5, 0.9. Each simulated dataset was spatially smoothed with a Gaussian filter. The FWHM of the Gaussian kernel is changed from 0.5 to 3 pixels.

### Real fMRI experiment

#### Participants

One right-handed college participant (age = 23) with normal vision was recruited. The subject was asked simply to relax with eye closed and remain still for 314 seconds during the whole fMRI scanning.

#### Imaging Parameters

Brain scans were performed at the MRI Center of Beijing Normal University using a 3.0-T Siemens whole-body MRI scanner. A single-shot T2*-weighted gradient-echo, EPI sequence was used for the functional imaging acquisition, with the parameters: TR/TE/flip angle = 2000 ms/30 ms/90o, FOV = 200×200 mm, matrix = 64×64, and slice thickness = 3.6 mm. 33 axial slices parallel to the AC-PC line were obtained in an interleaved order to cover the whole cerebrum and partial cerebellum. The anatomical MRI was acquired using a T1-weighted 128 slice MPRAGE sequence parallel to the sagittal plane which covers the whole brain. The parameters for this sequence were: TR/TE/flip angle = 2530 ms/3.39 ms/7°, FOV = 256×256 mm, matrix = 256×256, and slice thickness = 1.33 mm.

#### Preprocessing

Data were preprocessed using SPM2 software (Statistical Parametric Mapping; http://www.fil.ion.ucl.ac.uk/spm2). All functional images were realigned and spatially normalized into the standard MNI template space, resliced to 3×3×4 mm voxels.

#### Dimension Estimation

In order to investigate the performance of ITC in the real resting fMRI data with different Gaussian white noise levels, colored noise levels (including varied AR(1) coefficients and different SD levels of AR(1) noise), temporal data sizes and spatial smoothness, some new datasets were generated based on the preprocessed data before ITCs were applied. Firstly, four datasets with four different Gaussian white noise levels were generated by adding the additional Gaussian noise with SD equal to 0, 1, 2 and 3 to each voxel of the resting fMRI data. Secondly, three datasets with three different AR(1) coefficients were created by adding additional AR(1) noise with q equal to 0.1, 0.5 and 0.9. The SD of the three datasets was set to 2. Thirdly, three datasets with three SD levels of AR(1) noise were produced by adding additional AR(1) noise with SD equal to 1, 2 and 3 respectively. The q of the three datasets was set to 0.5. Fourthly, four datasets with varied temporal data sizes are comprised of the full preprocessed dataset with a temporal size of 157 and three other truncated datasets using only the first 100, 120 and 140 scans. Lastly, three datasets with different spatial smoothness includes two datasets spatially smoothed with a 4×4×4 mm^3^ and 8×8×8 mm^3^ Gaussian kernel and the unsmoothed dataset. ITCs were applied to each dataset.

## Results

### Simulations

#### White Noise model

**Figure 2:**
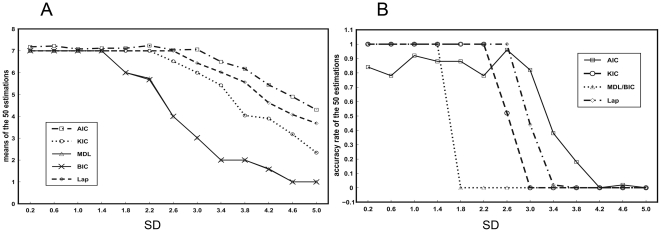
Results of the simulated data with varied Gaussian white noise levels. A) Means of the 50 estimations versus SD of Gaussian noises. B) Accuracy rate versus SD of Gaussian noises. The curves in the figures represent the estimation of different criteria as is specified in the legend.

The mean and accuracy rate of the 50 estimations of different ITCs versus the Gaussian noise level (SD) are shown in Fig. 2. Although all the criteria underestimate the true number of components at high noise level, MDL/BIC shows the severest underestimation and AIC exhibits the slightest underestimation (See Fig. 2A). For the low noise level, AIC is more likely to overestimate than the others (also See Fig. 2A). Except AIC, the accuracy rate of the other criteria are equal to 1 at low noise level. Moreover, for KIC, MDL, BIC and Laplace approximation, the accuracy rate of Laplace approximation decreases to zeros at relatively higher noise level and that of MDL/BIC decreases to zeros at relatively lower noise level. However, the accuracy rate of AIC decreases slowest at high noise level.

**Figure 3:**
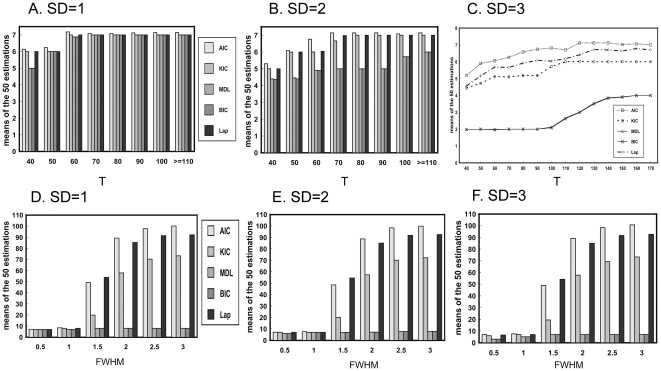
The variation of means of the 50 estimations with the temporal data size(A–C) and FWHM of Gaussian filter(D–F) at three different white noise levels. A) SD = 1. B) SD = 2. C) SD = 3. D) SD = 1. E) SD = 2. F) SD = 3.

Fig. 3A–C shows the variation of the means of estimations with the temporal data size at the three Gaussian noise levels. The results of all methods reach stable at relatively smaller temporal size (N = 110) when the Gaussian noise level (SD = 1 and 2) is not very high (See Fig. 3A–B). Nonetheless, the results are stable at relatively larger temporal size (N = 140) when the Gaussian noise level (SD = 3) is high (See Fig. 3C). For all noise levels, the estimations of all the criteria are more and more approximate to the true value with the increasing of the temporal size and tend to underestimate with the decreasing of the temporal size. Furthermore, the impact of the temporal data size on the estimations becomes more and more remarkable with the increasing of the Gaussian noise level. Among all the criteria, the MDL/BIC tends to yield the severest underestimation, especially at the high noise level.

Fig. 3D–F displays the mean estimation of all the criteria versus FWHM at the three Gaussian noise levels. Spatial smoothing may have pretty slight impact on the performance of all the criteria when the FWHM of Gaussian filter is smaller than one pixel. However, AIC, KIC and Laplace approximation show severe overestimation when the FWHM is larger than one pixel. The overestimations by the three criteria are raised with the increase of the FWHM. Compared to AIC, KIC and Laplace approximation, MDL/BIC manifests better performance even for high FWHM although it also has slight overestimation.

#### AR (1) noise model

**Figure 4:**
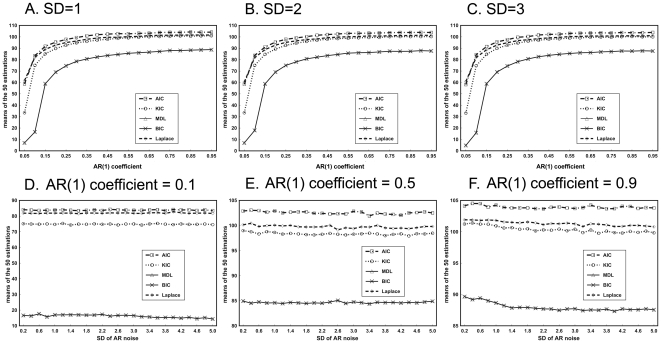
Results of simulated data with varied AR(1) noise levels. A–C) Means of the 50 estimations versus AR(1) coefficient at three levels of SD of AR(1) noise. D–F) Means of the 50 estimations versus SD of AR(1) noise at three AR(1) correlation levels.

Fig. 4 illustrates the changes of mean estimation of all criteria with the increase of AR(1) coefficient and SD of AR(1) noise. All of the criteria overestimate the number of components when the noise is temporally correlated. When q is less than 0.5, the estimation of ITCs rises rapidly with the increase of q. However, the estimation rises much slower when q is larger than 0.5 (See Fig. 4A–C). Moreover, all estimations vary slightly as SD of AR(1) noise increases (See Fig. 4D–F). Among all the criteria, MDL/BIC shows the least overestimation whereas AIC shows the most.

**Figure 5:**
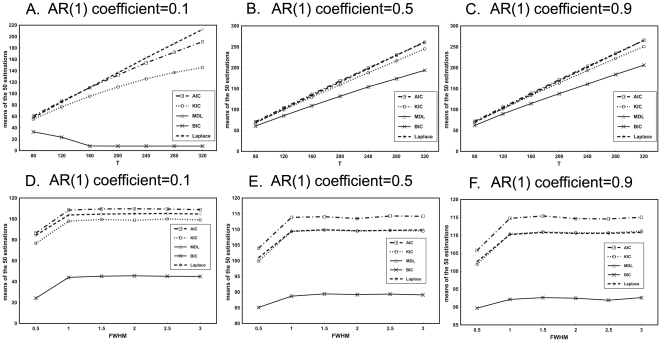
Results of the simulations with varied temporal data size (A–C) and different FWHM (D–F) for three AR(1) coefficient levels. A) SD = 1. B) SD = 2. C) SD = 3. D) SD = 1. E) SD = 2. F) SD = 3.

The variations of the means of estimations with the temporal data size at the three AR(1) coefficient levels are displayed in Fig. 5A–C. For all the three AR(1) coefficient levels, estimations of AIC, KIC and Laplace criteria increase with the rising of T. However, BIC/MDL tends to decline as T increases in the case of q = 0.1 and ascend in the case of q = 0.5 and 0.9.

The mean of the 50 estimations of different ITC versus FWHM are shown in Fig. 5D–F. When FWHM is smaller than or equal to 1 pixel, larger FWHM leads to larger estimations for all criteria. However, the estimations of ITCs exhibit very slight variation when FWHM is larger than 1 pixel. Moreover, BIC/MDL shows the slightest overestimation.

### Real fMRI experiment

**Figure 6:**
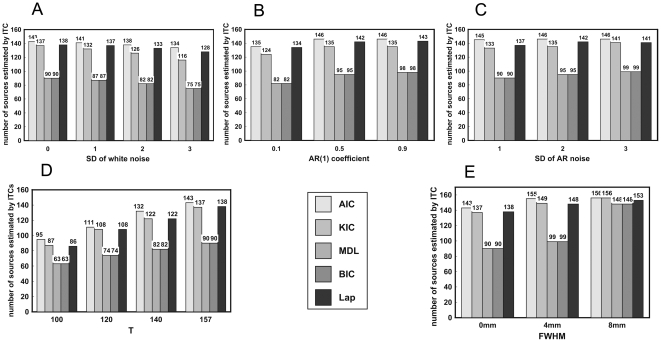
Results of the real resting fMRI data. A) Estimations of fMRI data with different added Gaussian noise levels. B) Estimations of fMRI data with different added AR(1) coefficient levels (SD = 2). C) Estimations of fMRI data with different added SD of AR(1) noise (q = 0.5). D) Estimations of data with different temporal data size. E) Estimations of data spatially smoothed by Gaussian filter with different FWHM.

Fig. 6 illustrates the results of real fMRI experiment. In Fig. 6A, the estimations of all the criteria reduce with the increase of the additionally added white noise level. Compared to the original fMRI data, the estimation of ITCs falls slightly when additional AR(1) noise with q = 0.1 is added. The estimation rises with the increase of q (See Fig. 6B). Fig. 6C shows that raising SD of AR(1) noise will slightly increase the estimation. Moreover, raising either the temporal size or the FWHM of Gaussian kernel leads to the increase of intrinsic dimension estimation for all the criteria (See Fig. 6D–E). In most cases, BIC/MDL produces the smallest estimations whereas AIC yields the largest estimations. These results of the real fMRI data are consistent with the above simulations.

## Discussion

In order to better understand the behavior of ITC, the performance of AIC, KIC, MDL, BIC and Laplace approximation were compared empirically through use of a variety of simulations with different Gaussian white noise levels, AR(1) noise levels, temporal data sizes and spatial smoothness. A number of conclusions were drawn, both about ITC in general, and about the performance of individual criterion in particular. Moreover, all the criteria were applied to the real resting fMRI data to further verify the findings of the simulations.

Some conclusions drawn from the results are consistent with previous studies. For instance, AIC is demonstrated to be an inconsistent estimator and tends to over-estimate at low Gaussian white noise level [16]. Despite inconsistency, our simulation based on white noise model demonstrated that AIC performs better at the high Gaussian noise level in contrast to the others [17]. BIC and MDL yield almost the same estimations in the simulated data and real fMRI data because the sample size of the simulation (N = 40000) and real fMRI data (N = 116865) in the current study are sufficiently large [16]. All the criteria will overestimate the number of components when the temporal correlation in noise cannot be neglected [24]. Spatial smoothing may lead to the overestimation [9], especially for AIC, KIC and Laplace approximation.

**Figure 7:**
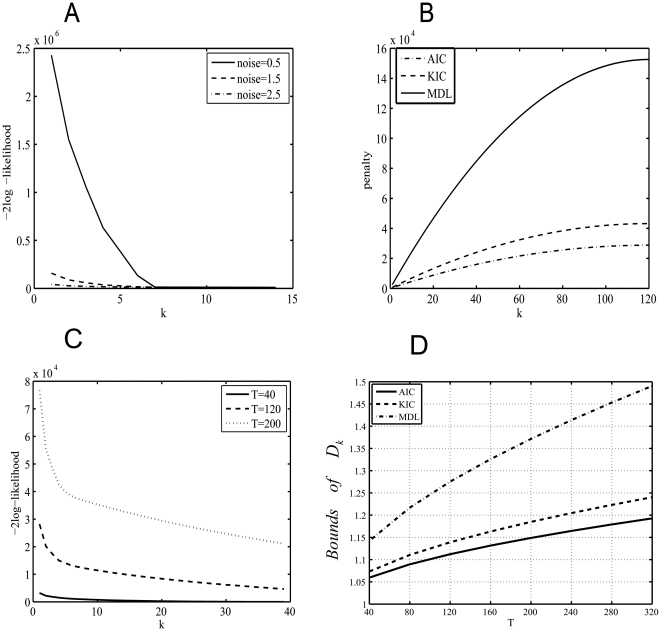
The log and penalty terms versus candidate order k and the bound BD versus the temporal data size. A) The variation of the negative log-likelihood with k at different noise levels. B) The penalty terms of AIC, KIC and MDL versus k. C) The variation of the negative log-likelihood with candidate order k at different temporal data size with SD = 1. D) The variation of the bound *BD* versus the temporal data size.

In terms of the pure white noise model, results show that high Gaussian noises can result in the underestimation of all the criteria. Moreover, Laplace approximation has the best robustness to the noises among the consistent estimators because its estimation accuracy rate decreases at the relatively high noise level. Although the formulas for AIC, KIC and MDL all have similar structures including the maximum log-likelihood term and the penalty term (See Eqs. (2, 3 and 6)), only the log term will vary with the noise level due to the impact of the noise on the distribution of the eigenvalues of the sample covariance matrix. Fig. 7A exhibits the variation of the first term of the formulas (−2Log-likelihood) with the candidate order k at different Gaussian white noise levels. It is observed that the decreasing rate of negative Log-likelihood reduces rapidly with the increasing of the Gaussian white noise level. The formula of AIC, KIC and MDL will reach minimum when the decreasing rate of negative Log-likelihood is equal to the increasing rate of the penalty. That means slower increasing rate of penalty is needed to adjust the negative Log-likelihood to ensure the correctness of estimation at the high Gaussian white noise level. However, the penalty of AIC, KIC and MDL is independent of the Gaussian noise level. Therefore, when adding the penalty having faster increasing rate to the negative Log-likelihood at high Gaussian noise level, the formulas are more likely to reach minimum earlier than the true value. Among the three criteria, the penalty of AIC grows slowest whereas that of MDL grows fastest with the increasing of k (See Fig. 7B) which may result in the least underestimation of AIC and the most underestimation of MDL. Moreover, it should be noted that the very small growing rate of AIC penalty is more likely to induce the overestimation of AIC at the low Gaussian noise level.

Next, smaller temporal data size also induces the underestimation of all the criteria because the decreasing rate of the negative Log-likelihood reduces fast with the decreasing of the temporal size T (See Fig. 7C). Although the growing rate of the penalty also reduces slowly with the decreasing of T, its reduction speed is much smaller compared to that of the negative Log-likelihood. Thus, the underestimation of small temporal size is severer in contrast to that of the large size. Moreover, large temporal size facilitates more accurate estimation of ITC at high Gaussian noise level because the fast decreasing rate of the negative Log-likelihood for large temporal size may counteract the slow decreasing rate of the negative Log-likelihood induced by high noise level.

Regarding the AR(1) noise model, it was found that bigger AR(1) coefficient q could result in more overestimation of ITCs although the rising speed of overestimation was largely reduced in the case of larger q (See Fig. 4A–C). By contrast, the increase of SD of AR(1) noise has very slight impact on the degree of overestimation (See Fig. 4D–F). From the AR(1) noise model in Eq.(13), we can see that bigger q indicates stronger autocorrelation of the colored noise. It has been reported that the break between signal eigenvalues and noise eigenvalues can be smoothed by the autocorrelations in colored noise [24]. Moreover, the dispersion of the noise eigenvalues will lead to overestimation because ITCs may ignore an arbitrarily large gap between the signal and the noise eigenvalues [20]. Therefore, it is the AR(1) coefficient rather than the SD of AR(1) noise that has more impact on overestimation, which is demonstrated by the results of the simulated data.

**Figure 8:**
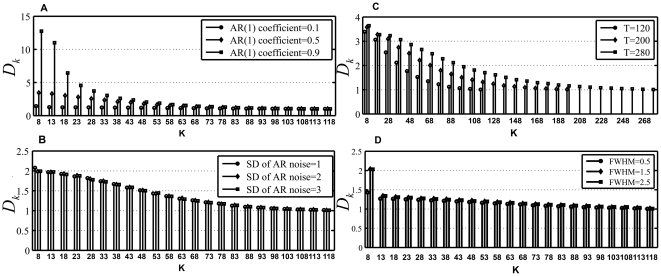
The variation of *D_k_* versus candidate order k. A) Different AR(1) coefficient levels. B) Different SD of AR(1) noise. C) Different temporal data size. D) Different FWHM.

Although all the criteria tend to overestimate in the case of AR(1) noise, AIC shows the severest overestimation and MDL/BIC shows the slightest overestimation. For the simulated data consisting of seven signals with the temporal data size T, the signal eigenvalues should be λ_1_, …, λ_7_ and the noise eigenvalues should be λ_8_, …, λ_T_. Liavas et al (2001) defined the metric 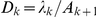 that represents the degree of the eigenvalue 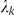 close to the last T-*k* eigenvalues λ*_k_*_+1_, …, λ_T_ [20]. 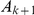 is arithmetic mean of the last T-*k* eigenvalues. Smaller 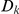 indicates that 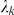 is closer to the last T-*k* eigenvalues.
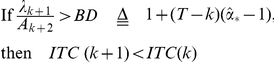
Here 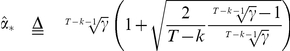 and 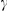 varied among AIC, KIC and MDL as listed below,
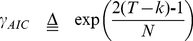
(14)

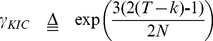
(15)

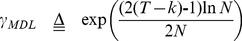
(16)The variable *BD* is a bound that can separate the eigenvalues of signals from those of the noise. In other words, the eigenvalues λ*_k_*_+1_, …, λ_T_ can be identified as noise eigenvalues only for *D_k_*>*BD* and *D_k+1_*<*BD*. Moreover, larger *BD* will make *D_k+1_* less than *BD* in the case of smaller k. Fig. 7D displays the variation of *BD* with temporal data size for AIC, KIC and MDL. It can be seen that MDL has the largest bound and AIC has the smallest bound. Thus, among all the criteria, the overestimation of MDL/BIC is the least and that of AIC is the most. In order to further examine the impact of q and SD of AR(1) noise on the dispersion of noise eigenvaules, the variations of *D_k_* with *k* (*k*>8) for different q and different SD of AR(1) noise are shown in Fig. 8A–B. Because *D_k_* increases rapidly with the increase of the AR(1) coefficient q for smaller k (See Fig. 8A), it can be inferred that the noise eigenvalues are clustered more closely in the case of smaller q. Moreover, *D_k_* will become less than the bound *BD* earlier for small q compared to large q. Therefore, small q contributes to less overestimation of ITCs relative to large q and overestimation becomes more likely for increasing the dispersion of noise eigenvalues. It should be noted that the increase of SD of AR(1) noise only lead to pretty slight variation of *D_k_* (See Fig. 8B). This indicates that the variation of SD of AR(1) noise does not affect the dispersion of noise eigenvalues.

Larger temporal data size can lead to more overestimation for AR(1) noise because increasing the temporal data size will raise *D_k_* and intensify the dispersion of noise (See Fig. 8C). Meanwhile, we also find that MDL/BIC exhibits different behavior from the other criteria when q is equal to 0.1. The estimation of MDL/BIC decreases with the increase of T and gradually approaches the true number of components. In contrast to the other criteria, the bound of MDL/BIC is the largest and manifests the fastest increasing speed with the raise of T (See Fig. 7D). Moreover, *D_k_* reduces with the decrease of q. Because the bound of MDL/BIC increases rapidly with T and small q counteracts the increasing of *D_k_* with the rising of T, the estimation of MDL/BIC lessens with the increase T in the case of q = 0.1.

Moreover, spatial smoothness intensifies the overestimation of ITCs in the case of AR(1) noise. However, the overestimation is not increased with FWHM when FWHM is larger than 1. Fig. 8D depicts the variation of *D_k_* with different FWHM. It can be seen that the head values of *D_k_* are much smaller for FWHM = 0.5 than FWHM = 1.5/2.5. However, there is only very small difference of *D_k_* between FWHM = 1.5 and FWHM = 2.5. Therefore, the simulated results of spatial smoothness can be interpreted by the variation of *D_k_* with FWHM.

Finally, it should be noted that the impact of SD of AR(1) noise on the real fMRI data is different from that of simulated data although most of results of the real fMRI data are in accordance with the simulated data. When only AR(1) noise exists in the simulated data, SD of AR(1) noise shows no impact on the degree of overestimation. However, larger SD of AR(1) noise induces larger estimation when AR(1) noise is added additionally to the real fMRI data. The different results may be attributed to the existence of white noise in the real fMRI data. Because large Gaussian white noise can result in underestimation, Gaussian white noise may counteract the o of AR(1) noise in the real fMRI data. However, the counteraction may become slighter and slighter and consequently the estimation rises gradually with the increase of SD of AR(1) noise.

In conclusion, Through both simulated and real fMRI data with varied Gaussian white noise levels, AR(1) noise levels, temporal data sizes and spatial smoothness, our study not only demonstrated some performances of ITC reported in the previous studies, but also obtained some additional conclusions regarding the performance of ITC. Based on the results of the current study, some suggestions on the selection of ITC to estimate the dimension of fMRI data were provided: 1) None of ITC is perfect for all fMRI data due to its complicated noise structure. 2) If there is only white noise in the data, AIC is preferred when the noise level is high and otherwise, Laplace approximation is a better choice. However, KIC may be better than Laplace approximation for the huge amounts of data at the low Gaussian noise level because Laplace approximation is more time consuming. 3) When colored noise exists in the data, MDL/BIC outperforms the other criteria.
